# Phytochemical Profile, Safety Assessment and Wound Healing Activity of *Artemisia absinthium* L.

**DOI:** 10.3390/plants9121744

**Published:** 2020-12-10

**Authors:** Amel Boudjelal, Antonella Smeriglio, Giovanna Ginestra, Marcella Denaro, Domenico Trombetta

**Affiliations:** 1 Department of Microbiology and Biochemistry, Faculty of Sciences, University of M’sila, M’sila 28000, Algeria; 2 Department of Chemical, Biological, Pharmaceutical and Environmental Sciences, University of Messina, Via Giovanni Palatucci, 98168 Messina, Italy; giovanna.ginestra@unime.it (G.G.); marcella.denaro@unime.it (M.D.); domenico.trombetta@unime.it (D.T.)

**Keywords:** *Artemisia absinthium* L., polyphenols, wound healing activity, antibacterial activity, acute dermal toxicity, acute oral toxicity

## Abstract

The aim of study was to validate, by in vitro and in vivo studies, the traditional use for wound-healing activity of *Artemisia absinthium* L. Reversed-phase liquid chromatography coupled with diode array detection and electrospray ion trap mass spectrometry (RP-LC-DAD-ESI-MS) analysis allowed to identify eleven polyphenols with chlorogenic acid as the most abundant compound (3.75 g/100 g of dry extract). After that, antibacterial activity as well as acute dermal and oral toxicity were assessed in animal models. In order to investigate the wound-healing activity of *A. absinthium* methanol extract, two ointments were formulated (MEO 5% and 10%). The ointment with the highest concentration of plant extract (10%) showed a statistically significant effect on the rats wound contraction, similar to that exerted by the reference drug Cicatryl-Bio. Moreover, *A. absinthium* methanol extract showed the best antibacterial activity against the Gram-negative *Escherichia coli* ATCC 10536 (MIC 1.25–2.5 mg/mL) and the Gram-positive *Staphylococcus aureus* ATCC 6538 (0.31–0.625 mg/mL). The absence of oral and topical toxicity of the treated animals allowed to establish the safety of the ointments. Overall, data collected in the present study support and validate the use of *A. absinthium* as a wound healing agent in the Algerian traditional medicine.

## 1. Introduction

There is a growing interest on the importance of medicinal plants and traditional practices in solving the world health care problems. Because of this awareness, the international trade in medicinal plants is growing exponentially and many countries have assimilated traditional medical practices as an integral part of their culture [1].

Algeria is one of the major African countries with a remarkable floristic richness, that is directly related to its ecosystem and landscape diversity. The number of taxa of its flora is estimated to be about 4000, including 300 endemic taxa of which approximately 90% are present in the north of the country [2]. Unfortunately, notwithstanding this large patrimony of Algerian flora, only few species have been deepen investigated.

Asteraceae represent one of the most important families of medicinal and aromatic plants growing in Algeria and comprises, among others, nine species of *Artemisia* [3].

Several ethnopharmacological studies have shown the use of plants belonging to the Asteraceae family as wound-healing agents. These evidences have been supported by several in vitro and in vivo studies and some of these plants such as *Calendula officinalis* L. and *Ageratina pichinchensis* (Kunth) R.M have been recently investigated also from the clinical point of view [4].

Moreover, some studies carried out also on isolated bioactive compounds such as silibinin (from *Silybum marianum* (L.) Gaertn.) and jaceosidin (from *Artemisia princeps* Pamp.), both belonging to the flavonoid’s class, shed lights on the promising use of these compounds for treatment of wounds, opening new perspectives for the development of new and efficient therapies [4].

*A. absinthium* is commonly called wormwood, and is locally known in Algeria as “Chajrat meriem” [5,6,7,8]. Various medicinal properties are recognized to *A. absinthium* in traditional medicine such as antidiabetic, antihypertensive, analgesic, antipyretic, antispasmodic, anti-inflammatory, and memory-improving proprieties as well as wound healing and skin-preserving properties [5,6,7,8].

Due the ethnobotanical importance of *A. absinthium* in Algeria and the lack of studies that validated the wound-healing potential of this plant, the aim of the present study has been to evaluate the wound-healing properties of a methanol extract of aerial parts of *A. absinthium* as well as antibacterial activity and to establish its safety by testing the acute oral and dermal toxicity.

In addition, a preliminary phytochemical screening followed by a reversed-phase liquid chromatography coupled with diode array detection and electrospray ion trap mass spectrometry (RP-LC-DAD-ESI-MS) analysis allowed to correlate the biological properties found with the most abundant bioactive compounds in order to support its traditional use.

## 2. Results

### 2.1. Extraction Yield and Phytochemical Characterization

The extraction yield of methanol extract from the flowering aerial parts of *A. absinthium* collected in Algeria was 20.85 ± 0.84% *w*/*w*. The preliminary phytochemical screening carried out by different in vitro assays, able to identify different class of compounds, showed that methanol extract contains a high amount of total phenols, 180.33 ± 16.25 mg gallic acid equivalents (GAE)/g of dry extract (DE) and flavonoids, 165.47 ± 13.32 mg rutin equivalents (RE)/g DE. Moreover, the proanthocyanidin and vanillin index determination allow to predict, by calculating the polimerization index (0.26), a high content of monomeric molecules (Table 1).

Reversed-phase liquid chromatography coupled with diode array detection and electrospray ion trap mass spectrometry (RP-LC-DAD-ESI-MS) analysis confirms what mentioned above and revealed the presence of 11 main compounds with the predominance of phenolic acids (Table 2), with chlorogenic acid as the most abundant compound (3.753 g/100 g DE).

**Figure 1:**
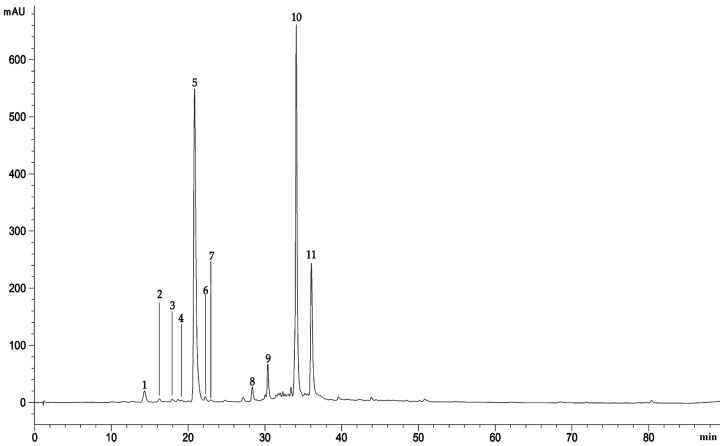
Representative LC-DAD chromatogram of methanol extract of aerial parts of *A. absinthium* acquired at 330 nm. Peak numbers refer to compounds listed in Table 2 according to their elution order.

The acquisition wavelength chosen to show the polyphenol profile of *A. absinthium* extract, at which all the identified peaks are visible, was 330 nm (Figure 1).

Apart the chlorogenic acid, the RP-LC-DAD-ESI-MS analysis revealed the presence of others abundant phenolic acids such as the dicaffeoylquinic acid and its derivative (31.60 and 13.38%, respectively), followed by 4-caffeoylquinic acid (1.65%), flavonoids such as the Luteolin-6-C-[β-D-glucosyl-(1->2)-α-L-arabinoside (1.40%) and chalcones such as 2′,6′-dihydroxy-4-methoxychalcone-4’-O-neohesperidoside (1.42%), among the most abundant compounds. Total identified compounds represent the 87.71%, with the remaining unidentified peaks which, as can be seen from the chromatogram showed in Figure 1, represent minor compounds with a mean area ≤ 0.2%.

### 2.2. Toxicity Tests

#### 2.2.1. Acute Dermal Irritation

Animals were observed frequently during the 14 days following the topical application of 0.5 g of 5% or 10% methanol extract ointments (MEO 5% and 10%). No sign of toxicity or mortality was observed. Rats maintain physiological conditions and did not show any critical changes in behavior and breathing, any disability in feeding and water utilization, postural irregularities or hair loss. Moreover, no signs of cutaneous irritation, erythema, eschar, edema, or any other skin reactions after topical application, were recorded.

#### 2.2.2. Acute Oral Toxicity

Acute oral toxicity was tested in mice at three different doses (1000, 2000, and 5000 mg/kg b.wt). No adverse effects such as respiratory, nervous, cutaneous and gastrointestinal symptoms or mortality were observed even at the highest administered dose.

Regarding biochemical parameters, the mice treated with the three doses showed no significant differences compared with the control group (Table 3).

The extract appeared to be well tolerated. Indeed, it did not affect nor liver and renal parameters in the treated mice compared with the control group (Table 3).

**Figure 2:**
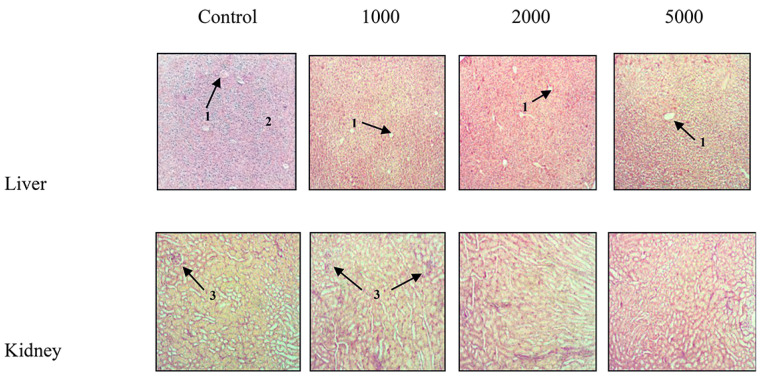
Histopathological changes in liver and kidney of animals treated with *A. absinthium* methanol extract at different doses (magnification 10×). 1: Central vein; 2: Surrounding hepatocytes; 3: Glomerulus.

Moreover, comparing the histological sections of liver and kidney of the treated mice with the respective controls, it is possible to observe that the extract administration did not induce any morphological change revealing a typical physiological kidney and hepatic lobule for all treated mice with respect to control (Figure 2).

### 2.3. Biological Properties

#### 2.3.1. Antibacterial Activity

MIC and MBC values of the methanol extract of *A. absinthium* with respect to the reference antibiotics against the investigated Gram-negative and Gram-positive bacteria were reported in Table 4.

Results showed that the methanol extract was able to inhibit the growth of the reference microorganisms with MIC values ranging from 0.31 to 2.5 mg/mL. The most sensitive strain was the Gram-positive *S. aureus* ATCC 6538 (MIC = 0.625–0.31 mg/mL) followed by the Gram-negative *E. coli* ATCC 10536 (MIC = 1.25–2.50 mg/mL). However, the methanol extract of *A. absinthium* is bacteriostatic against all the investigated bacterial strains at the tested concentrations. Negative control (1% DMSO) did not alter the microbial growth.

#### 2.3.2. Evaluation of the Wound Healing Activity

The wound-healing activity was evaluated measuring every 3 days the evolution of each wound excision in the treated and untreated animals. As shown in Table 5, there was a progressive daily decrease in the wound area throughout the experimental period in all the groups.

The treated animals (CIC, MEO 5%, MEO 10%, and PJ) showed a statistical significantly improve of the wound area in comparison with the untreated group (*p* < 0.001) (Figure S1). On the contrary, no significant difference was observed between the groups treated with the two concentrations of extract’s ointment (MEO 5% and 10%) and the reference drug Cicatryl-Bio, a pharmaceutical formulation containing 1% allanthoin able to improve and fasten reestablish the skin physiology. In particular, the MEO 10% was found to improve the wound contraction in rats in comparable manner with respect to the reference drug Cicatryl-Bio (81.49 ± 2.83% vs. 84.52 ± 0.80%, respectively). In the untreated group (negative control), the decrease in the wound area was not drastic as observed in the CIC-treated group (positive control). Indeed, the group treated with the reference drug showed a remarkable decrease in wound area between the 9th and 15th day with a superimposable behavior with respect to the other groups.

**Figure 3:**
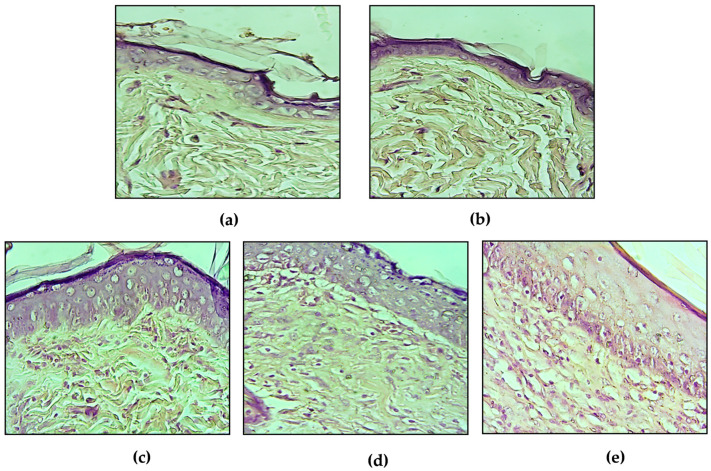
Histological evaluation of wound skin sections stained with hematoxylin and eosin (magnification 10×). Figure (**a**,**b**): Animals untreated (UT) and treated with petroleum jelly (PJ), respectively, showing fewer collagen fibers and plenty inflammatory cells. Figure (**c**–**e**): Animals treated with reference drug (CIC) and methanol extract ointments (MEO 5% and MEO 10% respectively) showing better healing with more collagen deposition and complete re-epithelialization.

At the end of the experiment, histopathological examination was carried out and results were depicted in Figure 3.

Histological evaluation of wound skin sections stained with hematoxylin and eosin showed the presence of fleshy bud with plenty inflammatory cells, less collagen deposition and incomplete maturation of the epidermis and dermis both in untreated (UT, Figure 3a) and petroleum jelly (PJ, Figure 3b) groups. Conversely, animals treated with reference drug (CIC, Figure 3c), MEO 5% (Figure 3d) and MEO 10% (Figure 3e) showed a better healing with more collagen deposition and complete re-epithelialization with thick mature epidermis and granulation tissue in comparison with the UT and PJ-treated animals.

## 3. Discussion

The TPC and TFC values obtained in this study fit perfectly within the range of total phenols and flavonoids determined in previous studies that have examined alcoholic extracts of *A. absinthium* from Tunisia (99.89 mg ± 3.30 GAE/g of DE and 126.40 ± 2.32 mg CE/g DW, for total phenols and flavonoids respectively), Romania (98.20 mg GAE/g DE) and Iran (194.0 ± 9.70 mg GAE/g of DE) [9,10,11].

According to our results, the most abundant polyphenol class identified in *A. absinthium* extract was that of phenolic acids (15.88–58.33% of the identified compounds), followed by flavones (9.98–38.61% of the identified compounds) [9]. However, the amount of each class of polyphenols identified as well as the relative abundance of individual constituents seem to vary greatly depending on the geographic characteristics of the place where the plants were collected [9]. The presence of apigenin, chlorogenic acid and two dicaffeoylquinic acid isomers have already been reported previously for *Artemisia* spp. [12]. Javed et al. [13], cited the presence of glycoside esters and glycoside isoflavones in the methanol extract of the aerial parts of *A. absinthium*. Moreover, a very recently review, which analyzed the latest advances in phytochemistry about the polyphenol profile of *A. absinthium*, refers not only to different phenolic acids (caffeoylquinic, dicaffeoylquinic, and chlorogenic acid) and flavonoids mentioned in the present study such as artemetin, apigenin, luteolin, and orientin, but also to calchones and organic acids such as cinnamic acid, in accordance with the present study in which a chalcone and a cinnamic acid derivative was found (1-*O*-β-D-glucopyranosyl sinapate and 2′,6′-dihydroxy-4-Methoxychalcone-4′-O-neohesperidoside, respectively) [14].

In our study, no sign of dermal toxicity was observed after application of the *A. absinthium* ointments. Moreover, after the oral administration, the methanol extract of *A. absinthium* did not produce any sign of toxicity or dead in mice even at the highest dose (5000 mg/kg b.w.) during and after 14 days of observation. In addition, no significant changes were observed in biochemical parameters or histological sections of liver or kidney except a slight dilation of the portal vein (Figure 2). Similar observations have been reported in the study of Daradka et al. [15,16] with ethanol extract (70%) of *A. abinthium* in rabbits and rats. According to the toxicity scale of Hodge and Sterner [17] for mice and rats, the extract of *A. absinthium* can be classified as a non-toxic substance [18]. Based on our data, short-term treatment with *A. absinthium* extract can be considered safe.

The absence of toxicity observed in our study may be explained by the relatively short treatment (2 weeks). Indeed, a 13-weeks repeated dose toxicity study of *A. absinthium* extract on both sexes of Wistar Hannover rats estimated a no-observed-adverse effect-level (NOAEL) of 1.27 g/kg/day and 2.06 g/kg/day in male and female rats, respectively [19].

In addition, also if the acute oral lethal dose 50 (LD_50_) in rats and the acute dermal LD_50_ in rabbits were reported as 960 mg/kg and 5 g/kg respectively, these parameters refer to *Artemisia* essential oil, which contains mainly thujone, a well-known toxic oxygenated monoterpene [20].

*A. absinthium* methanol extract showed a bacteriostatic behavior against all the bacterial strains investigated with the highest power against *S. aureus* ATCC 6538. This bacteriostatic potential may be due to the presence of the high level of chlorogenic acid and caffeoylquinic acids in the tested extract. Indeed, recent studies showed that chlorogenic acid is able to bind the outer bacterial membrane disrupting it, exhausting the intracellular potential and releasing the cytoplasm macromolecules, leading to the cell death [21]. In addition, Fiamegos et al. [22], identified caffeoylquinic acids as potent efflux pump inhibitors in a wide panel of Gram-positive human pathogenic bacteria.

The ointments containing the methanol extract of *A. absinthium* (MEO 5% and MEO 10%) significantly improved the wound healing process after excision in Wistar albino rats with respect to the untreated group. Despite a discrete wound contraction rate was observed also in the group treated with petroleum jelly, it does not have any therapeutic properties and keeping the wound moist, it prevents an adequate healing due to the maceration phenomenon, which flattens the contraction rate during time [23]. Based on histological examination, the treated groups (Cicatryl-Bio, MEO 5%, and MEO 10%) showed higher collagen deposition and complete re-epithelialization suggesting a similar wound healing mechanism with respect to allanthoin, the bioactive compound of Cicatryl-Bio used as reference drug, which occurs via the regulation of inflammatory response and stimulus to fibroblastic proliferation and extracellular matrix synthesis [24].

The best results were obtained with the highest dose of plant extract ointment (MEO 10%), which had a strong impact on the granulation and epithelialization of wounds, accelerating tissue repair and suggesting a dose–response relationship.

Chlorogenic acid is a phenolic acid well-known to accelerate the wound healing promoting epithelialization, fibroblast proliferation, and collagen formation and decreasing polymorph nuclear leukocytes infiltration [25,26]. However, despite a predominant role of chlorogenic acid is conceivable in the biological effects observed in the present study, a synergistic effect due to the presence of other phenolic acids and flavonoids cannot certainly be excluded.

It has been already demonstrated that other polyphenol extracts from leaves or flowering tops of plants belonging to the Asteraceae family act as promising wound-healing agents [4].

A flavonoid-rich leaf extract from *B. balsamifera* (L.) DC. showed wound contraction, capillary regeneration, collagen deposition, and re-epithelization following one week-treatment on Sprague–Dawley rats. These effects have been associated with an enhanced expression of several markers such as vascular endothelial growth factor, transforming growth factor-β1, and CD68 antigen in wound tissues. The main bioactive compounds responsible for these biological activity seems to be flavonoid aglycons, flavonoid glycosides, chlorogenic acid analogs, and coumarins [27]. Likewise, a polyphenol extract from flowering tops of *Achyrocline alata* (Kunth) DC. was found to accelerate the wound closure on mice, decreasing the local inflammation and promoting the re-epithelization and granulation of the injured tissues [28]. Moreover, recently, it has been demonstrated that leaf methanol extract from *A. absinthium* L. showed better wound healing properties than standard povidone iodine cream on rats, modulating several cytokine networks and apoptosis markers [29].

## 4. Materials and Methods

### 4.1. Plant Material and Extraction

The flowering aerial parts of *A. absinthium* were collected in May 2018, from M’sila province of Algeria (35°42′21″ N and 4°32′31″ E). The plant was identified and authenticated taxonomically by Sarri D. (Department of Nature Sciences and Life, University of M’sila, M’sila, Alegria). A voucher specimen of the plant (AB-13) was deposited in the herbarium of the same Department. The plant material was rinsed and air-dried in the shade at room temperature (RT) and finely powdered. The plant extract was obtained by a Soxhlet extraction. Specifically, powdered plant material (50 g) was treated with 500 mL of 85% methanol for 6 h. The mixture was then filtered on Whatman paper No.1 (GE Healthcare Europe GmbH, Milan, Italy) and the filtrate evaporated to dryness by a rotary evaporator (Buchi R-205, Cornaredo, Italy) at 37 °C.

### 4.2. Ointments Preparation

Topical formulations were obtained by incorporating the methanol extract of *A. absinthium* with petroleum jelly (PJ) (Unilever, France) at concentration of 5% and 10% (MEO 5% and 10%, respectively), which reflect the traditional preparation used by local herbalists to treat wounds [6]. Cicatryl-Bio (CIC), an allanthoin-based pharmaceutical preparation (Pierre Fabre, Paris, France), was used as reference drug.

### 4.3. Animals

All animals (Swiss albino mice weighing 30–33 g and Wistar albino rats weighing 200–220 g) were obtained from Pasteur Institute (Algiers, Algeria). They were fed ad libitum with water and kibble diet and housed in controlled conditions (temperature 22 ± 2 °C; relative humidity 60 ± 4%, in a 12-h light/dark cycle). Animal studies have been authorized by the Institutional Ethic Committee (Registration N°: DO1N01UN280120150001) and all procedures were performed according to the International Council for Laboratory Animal Science.

### 4.4. Phytochemical Studies

#### 4.4.1. Total Phenols Content

The total phenols content of the methanol extract was estimated by the Folin–Ciocalteu method according to Smeriglio et al. [30]. Briefly, 50μL of sample solution (0.187–3.0 mg/mL) was added to the Folin–Ciocalteu reagent (500 μL) followed by deionized water (450 μL). After 3 min, sodium carbonate (500 μL, 10% *w*/*v*) was added; samples were left in the dark at RT for 1 h mixing every 10 min, and the absorbance recorded at 785 nm, using an UV–Vis spectrophotometer (Shimadzu UV-1601, Kyoto, Japan). Gallic acid was used as the reference compound (0.075–0.60 mg/mL) and results were expressed as mg GAE/g DE.

#### 4.4.2. Total Flavonoids Content

The total flavonoids content was determined according to Lenucci et al. [31]. Briefly, 50 µL of sample solution (0.187–3.0 mg/mL) were mixed with 450 µL of distilled water, 30 µL of 5% NaNO_2_ and incubated for 5 min at RT. After that, 60 µL of 10% AlCl_3_ were added and sample incubated again for 6 min at RT. At the end of incubation time, 200 µL di 1 M NaOH and 210 µL of distilled water were added to the reaction mixture. The absorbance was recorded at 510 nm by an UV–Vis spectrophotometer (Shimadzu UV-1601, Kyoto, Japan). Rutin was used as reference compound (0.125–1.0 mg/mL) and results were expressed as mg RE/g DE.

#### 4.4.3. Vanillin Index Determination

The vanillin index was determined according to Barreca et al. [32]. Briefly, 2.0 mL of sample solution was added with 0.5 M H_2_SO_4_ (absorbance unit 0.2–0.4), were loaded onto a conditioned SepPak C18 cartridge (Waters, Milan, Italy). The column was washed with 2.0 mL of 5.0 mM H_2_SO_4_ and purged with air, and the sample eluted with 5.0 mL of methanol. One milliliter of the methanol eluate was placed in a test tube (shielded from light), together with 6.0 mL of 4% vanillin methanol solution and immersed in a water bath at 20 °C for 10 min. Three milliliters of 37% HCl were added and after 15 min, the absorbance was recorded at 500 nm by an UV–Vis spectrophotometer (Shimadzu UV-1601, Kyoto, Japan). Catechin was used as reference compound (31.25–500 μg/mL) and results were expressed as mg CE/g DE.

#### 4.4.4. Proanthocyanidins Content

The proanthocyanidins content was determined according to Barreca et al. [32]. Briefly, sample diluted 10 times with 0.05 M H_2_SO_4_ in order to obtain a final volume of 2.0 mL was loaded onto a conditioned Sep-Pak C18 cartridge (Waters, Milan, Italy), pre-conditioned with 2.0 mL of 5 mM H_2_SO_4_ and purged with air. The proanthocyanins-rich fraction was eluted with 3.0 mL of methanol and collected in a 100 mL round-bottom flask containing 9.5 mL of absolute ethanol, shielded from light. After that, 12.5 mL of FeSO_4_•7H_2_O solubilized in 37% HCl (300 mg/L) was added and the round-bottom flask was placed in a boiling water bath and refluxed for 50 min. Sample was then rapidly cooled by immersion in cold water (20 °C) and after 10 min, the absorbance was recorded at 550 nm by an UV–Vis spectrophotometer (Shimadzu UV-1601, Kyoto, Japan). The basal anthocyanins content of sample was obtained detracting the absorbance of a sample prepared under the same conditions, but cooled in ice and not warmed. The proanthocyanidins content was conventionally expressed as 5 times the amount of cyanidin formed, by means of a calibration curve with cyanidin chloride (ɛ = 34,700). Results were expressed as mg CyE/g of DE.

#### 4.4.5. Polyphenol Profile Characterization

RP-LC-DAD-ESI-MS analysis was carried out by an Agilent 1100 series HPLC system (Santa Clara, CA, USA) using a Luna Omega PS C18 column (150 × 2.1 mm, 5 µm; Phenomenex, Torrance, CA, USA). Separation, was carried out at RT with a flow rate of 0.4 mL/min using a mobile phase consisting of solvent A (0.1% Formic acid) and solvent B (Acetonitrile) according to the following elution program: 0–3 min, 0% B; 3–9 min, 3% B; 9–24 min, 12% B; 24–30 min, 20% B; 30–33 min, 20% B; 33–43 min, 30% B; 43–63 min, 50% B; 63–66 min, 50% B; 66–76 min, 60% B; 76–81 min, 60% B; 81–86 min, 0% B; 86–90 min, 0% B. The injection volume was 5 µL. The UV-Vis spectra were recorded in the 190–600 nm range and chromatograms were acquired at 292 nm for hydroxycinnamic acids and flavanols, 330 nm for flavones and 370 nm for flavonols. The most abundant compound, chlorogenic acid, was quantified by using an external calibration curve built with the reference standard (Extrasynthese 4991 S, HPLC grade ≥ 99%).

The experimental parameters of the mass spectrometer (ion trap model 6320) operating in both positive (ESI+) and negative ionization (ESI−) mode were set as follows: The capillary voltage was 3.5 kV, the nebulizer (N_2_) pressure was 40 psi, the drying gas temperature was 350 °C, the drying gas flow was 9 L/min and the skimmer voltage was 40 V. The mass spectrometer was operated in full-scan mode in the m/z range 90–1000. Data were acquired by Agilent ChemStation version B.01.03 and Software Trap control version 6.2.

### 4.5. Toxicity Assays

#### 4.5.1. Acute Dermal Irritation

The acute dermal irritation assay was carried out on Wistar albino rats. The study was conducted according to the Organization for Economic Cooperation and Development (OECD) guidelines 404 [33]. Five-hundred (500) mg of each ointment (MEO 5% and 10%) were applied topically on the rats’ dorsal fur (3 mice/group). The number of animals has been chosen according to the International Guiding Principles for Biomedical Research Involving Animals recently revised by the International Council for Laboratory Animal Science (ICLAS) and Council of International Organizations of Medical Sciences (CIOMS) in order to use, according to the three R’ principle, the minimum number of animals necessary to achieve the scientific goal, refining the experimental techniques [34].The animals were observed for mortality and any toxic or deleterious effects with special attention given to the first 4 h and then once daily for a period of 14 days following the topical application. All animals were examined for skin reactions such as erythema and oedema as well as other local injury.

#### 4.5.2. Acute Oral Toxicity

The acute oral toxicity was assessed in healthy young adult female nulliparous and non-pregnant Swiss albino mice. The study was conducted according to the Organization for Economic Cooperation and Development (OECD) guidelines 423 [35]. The number of animals has been chosen according to the International Guiding Principles for Biomedical Research Involving Animals (see Section 4.5.1) [34]. Twelve Swiss albino mice were divided into four groups of three animals each one and treated orally with different doses of *A. absinthium* extract suspended in a vehicle (distilled water) for 14 days by gavage: Group I (control) received distilled water; Group II received 1000 mg/kg b.wt; Group III received 2000 mg/kg b.wt and Group IV received 5000 mg/kg b.wt. Following the fasting period (12 h), body weight of the mice was determined and the doses were calculated in reference to the body weight at 10 mL/kg. According to Abrar et al. [36], the physical aspect as well as the behavioral changes and clinical signs such as changes in heart and respiratory rate, abdominal contractions and diarrhea were evaluated.

At the end of the experiment, mice were sacrificed. Blood samples were collected to explore biochemical parameters (transaminases, alkaline phosphatase, serum total protein, creatinine, urea, and uric acid) by commercially available enzymatic kits (Spinreact, Girona, Spain) according to the manufacturing instructions. Moreover, liver and kidneys were recovered for histopathological examinations according to Gandhare et al. [37].

### 4.6. Biological Properties

#### 4.6.1. Antibacterial Activity

In many types of acute and chronic wounds, the impaired skin is frequently infected by typical microorganisms such as *Staphylococcus aureus, Staphylococcus epidermis, Pseudomonas aeruginosa,* and *Escherichia coli*; they are usually encountered growing simultaneously in co-cultures [38].

The methanol extract was screened for antibacterial activity by micro-dilution broth method according to the protocols recommended by the Clinical and Laboratory Standards Institute [39,40] using Mueller-Hinton broth (MHB; Oxoid, Milan, Italy) at 37 °C for 24 h.

Minimal inhibitory concentration (MIC) and minimum bactericidal concentration (MBC) of the tested extract (0.01–2.5 mg/mL) was evaluated by a wide panel of standard strains from the University of Messina’s in-house culture collection including Gram-negative (*Escherichia coli* ATCC 10,536 and *Pseudomonas aeruginosa* ATCC 9027) and Gram-positive bacteria (*Staphylococcus aureus* ATCC 6538 and *Staphylococcus epidermidis* ATCC 35984). Moreover, several clinical strains of *S. aureus* isolated by pharynges (526, 530, 808, 581), duodenal ulcers (8 and 14) and hip prostheses (3, 32, 84) were investigated. Results were expressed as mean of three independent experiments in triplicate (*n* = 3). Vancomycin and tobramycin were used as positive controls for Gram-positive and Gram-negative strains, respectively whereas 1% DMSO was used as negative control.

#### 4.6.2. Wound-Healing Activity

The rats were weighed, marked and divided in 7 groups of 5 rats. After that, their dorsal fur was shaved and they were left in their cages for 24 h to verify the absence of irritation in the shaved zone [41]. Animals were anaesthetized using an intraperitoneal injection of ketamine (90 mg/kg)-xylazine (10 mg/kg) according to Mashreghi et al. [42]. A circle wound (2.5 cm in diameter) was drawn on the skin of the lumbar region and was then excised [43]. Animals were then placed in individual cages with clean litters and the excisional wounds immediately measured. Five-hundred (500) mg/rat of CIC, MEO 5%, MEO 10% and PJ were applied topically once a day for 18 consecutive days [44]. Diameter of the excisional wounds was measured every 3 days by plotting the wounds on transparent paper and then measuring them using millimeter ruler. Percentage evolution of wounds contraction was calculated according to the following equation [45]:

% Wound contraction = [(Initial wound size − Specific day wound size)/Initial wound size] × 100

On day 19, the rats were euthanized and tissue samples recovered for histological evaluation. Tissues were fixed in formalin (10%) and embedded in paraffin. Sections of 5 µm were stained with haematoxylin/eosin according to Marque et al. [46] and examined by Optika B-500 microscope (Ponteranica, Bergamo, Italy).

### 4.7. Statistical Analysis

Results were expressed as mean ± standard deviation (SD). Significance between control and treated groups were determined by GraphPad Prism software (version 7.00, GraphPad Software, San Diego, CA, USA) using one-way analysis of variance (ANOVA), followed by Dunnett’s test. Differences were considered statistically significant at *p* < 0.05.

## 5. Conclusions

The topical formulation containing 10% *A. absinthium* extract (MEO 10%) showed significant antibacterial and wound healing properties also in comparison with the reference drug Cicatryl-Bio. Acute oral and dermal toxicity studies in albino mice and rats indicated that extract obtained from aerial parts of *A. absinthium* is safe over a two-week treatment period corresponding to a typical application time in the wounds therapy. Therefore, although further studies on the predominant bioactive compounds of the extract under consideration are needed, data of the present study confirm and scientifically validate the therapeutic application of *A. absinthium* extract for the treatment of wounds supporting the Algerian traditional use of this plant as a wound healing agent.

## Figures and Tables

**Table 1:** Phytochemical screening of methanol extract from aerial parts of *Artemisia absinthium*. Values were expressed as means ± SD (*n* = 3).

Assays	Methanol Extract
Total phenols (mg GAE ^1^/g DE ^2^)	180.33 ± 16.25
Total flavonoids (mg RE ^3^/g DE)	165.47 ± 13.32
Proanthocyanidyns (mg CyE ^4^/g DE)	43.11 ± 2.02
Vanillin index (mg CE ^5^/g DE)	11.17 ± 0.68
Polimerization index (VI ^6^/PC ^7^)	0.26

^1^ GAE, gallic acid equivalents; ^2^ DE, dry extract; ^3^ RE, rutin equivalents; ^4^ CyE, cyanidin chloride equivalents; ^5^ CE, catechin equivalents; ^6^ VI, Vanillin index; ^7^ PC, Proanthocyanidyns content.

**Table 2:** Phytochemical profile of *A. absinthium* aerial parts methanol extract by RP-LC-DAD-ESI-MS analysis. Results were expressed as mean relative area percentage of each compound with respect to the total compounds identified.

Compound	RT	λ_max_	[M-H]^-^	MW	Area%
4-Caffeoylquinic acid	14.323	232; 262; 300	353	354	1.65
Homorientin	16.261	232; 262; 300	447	448	0.35
5-Caffeoylquinic acid	17.909	232; 264; 312	353	354	0.30
Apigenin-7-*O*-glucoside	19.147	232; 264; 310	431	432	0.18
Chlorogenic acid	20.840	232; 264; 310	353	354	37.01
Artemetin	22.240	232; 264; 310	387	388	0.37
1-*O*-β-D-glucopyranosyl sinapate	22.986	232; 264; 310	385	386	0.05
2′,6′-dihydroxy-4-Methoxychalcone-4′-*O*-neohesperidoside	28.361	232; 270; 310	593	594	1.42
Luteolin-6-C-[β-D-glucosyl-(1->2)-α-L-arabinoside	30.715	232; 322	579	580	1.40
3,4-Dicaffeoylquinic acid	34.086	232; 294; 324	515	516	31.60
3,5-Dicaffeoylquinic acid	36.052	232; 296; 326	515	516	13.38
Total identified compounds					87.71

**Table 3:** Biochemical parameters evaluated in blood of mice treated with the methanol extract of *A. abinthium* at different doses. Values are expressed as means ± SD (*n* = 3). Data did not show any statistically significant difference in comparison with control group.

Parameters	Treatment (mg/kg b.wt)			
	Control	1000	2000	5000
Urea (mg/L)	37.31 ± 1.75	33.85 ± 1.52	32.99 ± 0.62	38.97 ± 4.14
Creatinine (mg/L)	4.09 ± 0.62	4.31 ± 0.64	4.77 ± 0.28	4.69 ± 0.40
Uric Acid (mg/dL)	38.57 ± 5.72	37.14 ± 3.46	34.76 ± 3.51	39.05 ± 3.33
AST (IU/L)	66.08 ± 6.61	67.96 ± 7.80	69.27 ± 6.10	72.63 ± 8.34
ALT (IU/L)	22.02 ± 3.95	17.65 ± 1.53	19.83 ± 0.86	26.54 ± 1.77
ALP (IU/L)	45.67 ± 5.84	56.58 ± 6.36	57.15 ± 8.09	59.34 ± 4.84
Total Protein (g/dL)	60.00 ± 2.64	56.12 ± 7.55	62.52 ± 3.70	52.38 ± 2.31

**Table 4:** Minimum inhibitory concentration (MIC) and minimum bactericidal concentration (MBC) values of methanol extract of *A. absinthium* and reference antibiotics (tobramycin and vancomycin) against the Gram-negative and Gram-positive bacteria. Results were expressed as mean of three independent experiments (*n* = 3).

Bacteria	Methanol Extract		Reference Antibiotics	
	MIC (mg/mL)	MBC (mg/mL)	MIC (µg/mL)	MBC (µg/mL)
Gram-negative			Tobramycin	
*Escherichia coli* ATCC 10536	2.5–1.255	>2.5	0.50	0.50
*Pseudomona aeruginosa* ATCC 9027	>2.5	>2.5	0.25	0.25
Gram-positive			Vancomycin	
*Staphylococcus aureus* ATCC 6538	0.625–0.31	>2.5	0.31–0.62	0.31–0.62
*Staphylococcus epidermidis* ATCC 35984	>2.5	>2.5		
*S. aureus* 526	>2.5	>2.5		
*S. aureus* 530	>2.5	>2.5		
*S. aureus* 808	>2.5	>2.5		
*S. aureus* 581	>2.5	>2.5		
*S. aureus* 8	>2.5	>2.5		
*S. aureus* 14	>2.5	>2.5		
*S. aureus* 3	>2.5	>2.5		
*S. aureus* 32	>2.5	>2.5		
*S. aureus* 84	>2.5	>2.5		

**Table 5:** Effect of different treatments on the evolution of the excision wounds contraction percentage (%) in Wistar albino rats during treatment period (18 days). Values are expressed as mean ± SD (*n* = 5).

Group	Wound Contraction % during Time (days)					
	3	6	9	12	15	18
UT	11.58 ± 1.67	17.82 ± 1.2	19.77 ± 2.83	20.54 ± 2.39	22.32 ± 2.3	27.54 ± 1.10
CIC	13.02 ± 0.96	29.81 ± 3.29 ***	49.91 ± 1.55 ***	70.53 ± 1.19 ***	75.87 ± 1.12 ***	84.52 ± 0.80 ***
MEO 5%	14.46 ± 2.01	30.27 ± 0.47 ***	43.18 ± 1.27 ***	57.59 ± 1.43 ***	68.41 ± 2.49 ***	76.73 ± 2.97 ***
MEO 10%	14.60 ± 0.59	25.75 ± 0.91 **	35.83 ± 1.51 ***	61.66 ± 1.24 ***	75.45 ± 1.70 ***	81.49 ± 2.83 ***
PJ	12.99 ± 2.27	24.79 ± 1.6 **	34.85 ± 1.61 ***	36.51 ± 1.37 ***	44.75 ± 1.85 ***	50.56 ± 1.52 ***

** *p* < 0.01, *** *p* < 0.001 vs. untreated group; UT: Untreated group; CIC: Group treated with Cicatryl-Bio containing 1% allanthoin; MEO 5%/10%: Groups treated with methanolic extract ointment; PJ: Group treated with petroleum jelly.

## Supplementary Materials

The following are available online at https://www.mdpi.com/2223-7747/9/12/1744/s1, Figure S1: Effect of different treatments on the evolution of the excision wounds contraction. UT: Untreated group; CIC: Group treated with Cicatryl-Bio containing 1% allanthoin; MEO 5%/10%: Groups treated with methanolic extract ointment; PJ: Group treated with petroleum jelly.
