# Supplementary figures and images for: Phytochemical Profile, Safety Assessment and Wound Healing Activity of Artemisia absinthium L
Source: Plants (Basel). 2020 Dec 10;9(12):1744. doi: 10.3390/plants9121744 (PMC7763807; doi:10.3390/plants9121744)

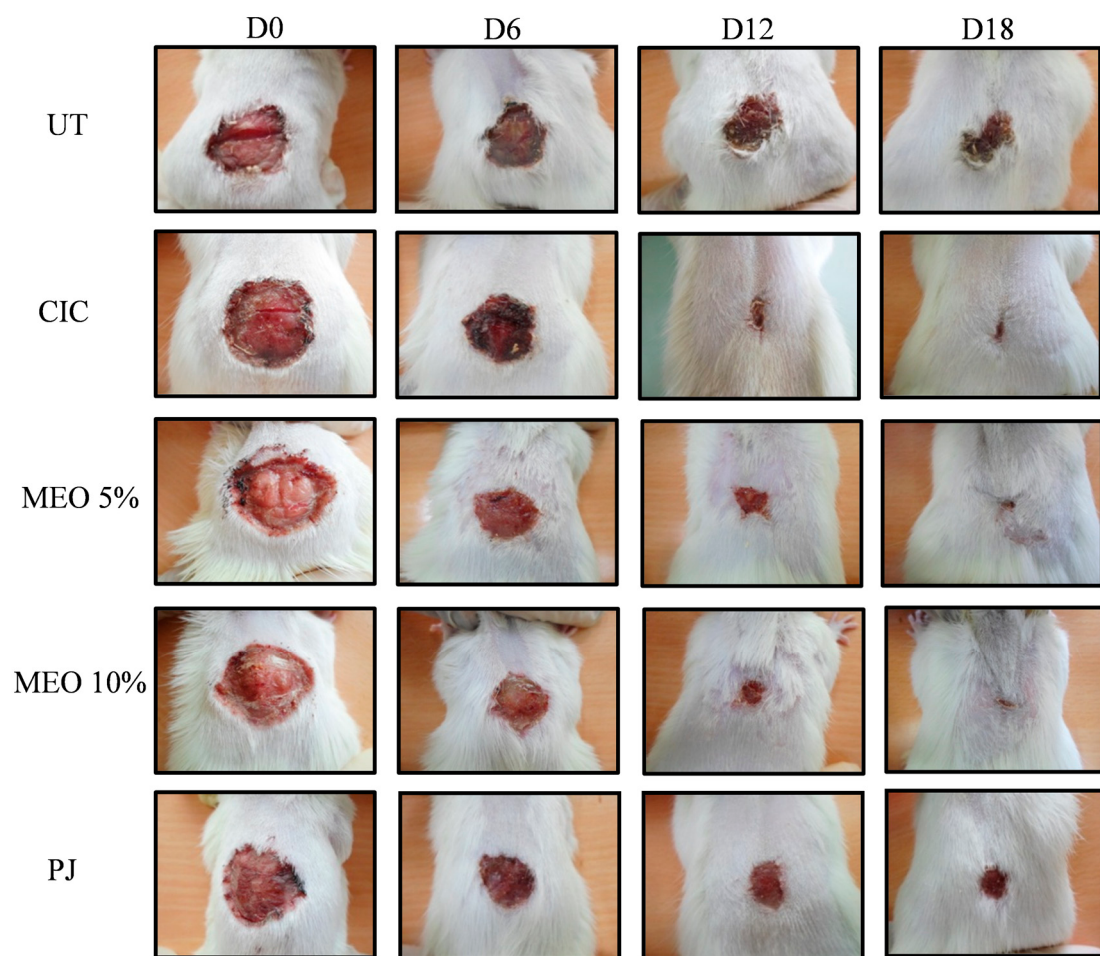

Supplement: Supplementary file 1 [file plants-09-01744-s001.pdf]
